# Non-functional paraganglioma of urinary bladder managed by transurethral resection

**DOI:** 10.1590/S1677-5538.IBJU.2018.0604

**Published:** 2019-01-29

**Authors:** Baomin Qiao, Baochao Zhang, Zhenrui Fu, Liwei Liu, Chunyu Liu

**Affiliations:** 1 Tianjin Institute of Urology Second Hospital Tianjin Medical University Tianjin China Department of Urology, Tianjin Institute of Urology, The Second Hospital of Tianjin Medical University, Tianjin, China

**Keywords:** Paraganglioma, Urinary Bladder, Transurethral Resection of Prostate

## Abstract

**Purpose:**

As a rare bladder tumor, paraganglioma of the urinary bladder (PUB) is frequently misdiagnosed as bladder cancer, particularly for the non-functional type. To date, transurethral resection remains a controversial treatment for non-functional PUB. This study aimed to identify the clinical features, pathological characteristics, prognosis, and safe/effective treatment of non-functional PUB using transurethral resection of the bladder tumor (TURBT).

**Materials and Methods:**

The clinical records, radiological data, pathological characteristics and follow-up times were retrospectively reviewed in 10 patients with clinically and pathologically proven non-functional PUB in our hospital from January 2008 to November 2016. All patients underwent TURBT treatment.

**Results:**

The incidence of non-functional PUB in patients with bladder cancer was 0.17%. The mean age at diagnosis was 44.5 ± 13.6 years (range, 29-70 years), and the patient population had a female: male ratio of 3: 2. No patients had excess catecholamine (CA) whilst four patients had painless hematuria. All neoplasms were completely resected via TURBT. The majority of samples were positive for immunohistochemical markers including chromogranin A (CgA) and Synaptophysin (Syn), but were negative for cytokeratins (CKs). Only a single recurrence was observed from the mean follow-up period of 36.4 ± 24.8 months.

**Conclusion:**

Complete TURBT is a safe and efficient treatment that serves both diagnostic and therapeutic purposes. Histopathological and immunohistochemistry examinations are mandatory for diagnostic confirmation. Long-term follow-up is recommended for patients with non-functional PUB.

## INTRODUCTION

Paraganglioma of the urinary bladder (PUB) is a rare type of bladder tumor that accounts for approximately 0.06% of bladder tumors, 1% of pheochromocytomas, and 10% of paragangliomas (extra-adrenal pheochromocytoma) (1). PUB is a neuroendocrine neoplasm, which arises from chromaffin cells located in the muscle layer of the bladder wall (2). The majority of PUB is solitary, arising on the dome or the trigone of bladder (3). Based on the content and activity of catecholamine (CA) that arise from the tumor, extra-adrenal paraganglioma can be classified as functional (chromaffin) or non-functional (non-chromaffin). The former clinically manifests as hematuria or catecholamine-related symptoms including micturition syncope, hypertension, headache, palpitations and transient hypertension after urination. The latter shows no obvious symptoms, but compression occurs when the tumor becomes large. In 1953, Zimmerman et al. (4) reported the first case of PUB. As the clinical presentation of PUB did not occur during the early disease stages, clinicians were unaware of the tumor, frequently leading to misdiagnosis or missed diagnosis, particularly for the non-functional type.

In this study, we report the clinical features, pathological characteristics, and prognosis of patients diagnosed as non-functional PUB treated by transurethral resection of the bladder tumor (TURBT) in our hospital. This enhances our knowledge and understanding of non-functional PUB and the safety and effectiveness of TURBT.

## MATERIALS AND METHODS

From January 2008 to November 2016, 10 patients were diagnosed as non-functional PUB according to postoperative pathologic reports at our hospital. These accounted for approximately 0.17% of all bladder tumors reported during the same term. We retrospectively reviewed the clinical records, operative notes, pathologic reports, and follow-up records of the patients. To evaluate both the position and clinical stage of the tumors, all patients received preoperative abdominal ultrasound, urine cytology and computed tomography (CT) examinations. Cystoscopy was performed in a single case. All neoplasms were completely resected by TURBT. During tumor resection, blood pressure, heart rate, and microcirculation status modestly changed. All surgical specimens were diagnosed by at least two urological pathologists. The pathological tumor stage was estimated according to the Cancer tumor-node-metastasis (TNM) staging system on bladder cancer. Long-term follow-up was performed to evaluate the therapeutic outcome of non-functional PUB. The mean follow-up period was 36.4 ± 24.8 months (range: 8-95 months).

## RESULTS

### Clinical Features

In this study, 10 cases of non-functional PUB were identified, accounting for 0.17% (10/5680) of patients with bladder cancer. In all patients, tumors were incidentally detected on imaging studies. The mean age of the non-functional PUB patients were 44.5 ± 13.6 years (range 29-70 years), the mean body mass index (BMI) was 23.5 ± 3.6, 4 patients were male and 6 were female, and the mean blood pressure of patients was ≤ 140/90mmHg. All patients had no symptoms of excess CA and four patients had painless hematuria. Tumor sizes ranged from 1.5cm x 1.3cm to 3.5cm x 2.1cm.

### Surgical treatment and Pathological findings

**Figure 1:**
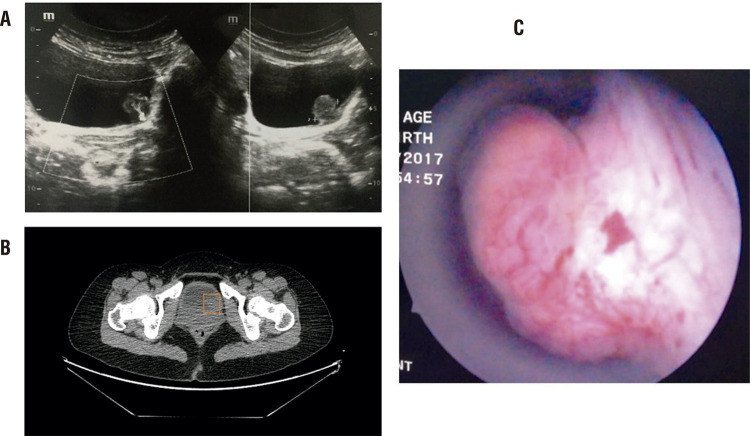
Imaging and cystoscopy. A) Ultrasound examination showing a hypoechoic mass with abundant blood flow inside in the urinary bladder. B) CT scan showing a polypoid mass (box) in the left wall of the urinary bladder. C) Cystoscopy showing solitary and broad basal neoplasms covered by smooth vesical mucosa in the left anterior wall of the urinary bladder.

**Figure 2:**
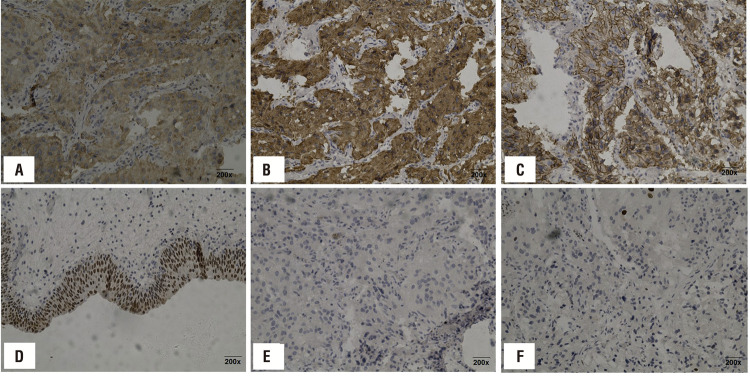
Immunohistochemistry. A) CgA; B) Syn; C) CD56; D) GATA3; E) CK7; F) Ki67; expressed in PUB cells (IHC 200x).

All patients underwent ultrasonography, urine cytology and CT examinations after hospitalization. Most PUBs were localized, solitary, spherical, broad basal tumors and extended towards the bladder cavity according to imaging films (Figures 1A and B). During cystoscopy (Figure-1C), neoplasms were covered by smooth vesical mucosa, with an abundant blood supply and calcification observed in two cases. TURBT was performed in all patients and all surgical margins were negative. The mean surgery time was 39.6 ± 6.7 minutes. All patients had a high tolerance for the operational procedures with no significant postoperative complications. From pathological macroscopic findings, the non-functional PUB specimens were daffodil or dark yellow. Microscopically, tumor tissues were comprised of a dense array that was divided into numerous small nests. Tumor cells were polygonal and fusiform with stippled chromatin and inconspicuous nucleoli. Immunohistochemistry revealed that the majority of tumor cells were positive for chromogranin A (CgA) and Synaptophysin (Syn), but negative for cytokeratin markers (CKs), including CK7 and CK20. Three tumors were positive for GATA3 and two were positive for CD56. The Ki-67 positive percentage of PUB cells was 1% to 30% (Figure-2A-F). According to the pathological reports, 7 cases were at stage T1, and 3 cases were at T2.

### Follow-up

All patients received no further treatment and were followed up with physical examinations, laboratory tests, and abdominal ultrasound or pelvic cavity CT and cystoscopy examination every 3-6 months, and then annually. The mean postoperative follow-up period was 36.4 ± 24.8 months (range, 8-95 months). Only one case of T2 relapsed on the 37th month and TURBT was again performed in this patient. A single patient died of myocardial infarction 8 months post-operation.

## DISCUSSION

Paraganglioma, also named extra-adrenal pheochromocytoma, is a rare tumor that is derived from chromaffin tissues of the sympathetic and parasympathetic nervous system (5). Paragangliomas are mostly located in the abdomen and pelvic cavity, whilst paraganglioma in the bladder is extremely rare, constituting ≤ 1% of all urinary bladder tumors, 6% of all paragangliomas (6). In additon, only 10% of PUB cases are malignant (1). To-date, the diagnosis of malignant paraganglioma relies on the invasiveness of the neoplasm; namely, the primary lesion metastasizes to non-chromaffin tissues or organs, including the lymph nodes, liver, spleen, and bone (7). Honma et al. (8) illustrated that paraganglia can occur in any position of the bladder wall, mostly in the anterior and posterior walls of bladder and is rarely seen in the bladder trigone.

CA is a neurogenic substance consisting of catechol and amino groups, including norepinephrine, adrenaline and dopamine, which are mainly secreted by chromaffin cells. The level of CA is often found to significantly increase in pheochromocytoma and functional paraganglioma due to the symptoms of paroxysmal hypertension. According to catecholamine secretion, PUB can be classified into functional and non-functional types. The majority of PUBs are functional groups characterized by excess catecholamines and symptoms, including paroxysmal hypertension, palpitation, micturition syncope, and headaches (5). In some cases, these features are absent. As such, patients are often misdiagnosed as urothelial cancer during pre-operative evaluations (9, 10). According to previous studies, 61.6% of PUB, confirmed by postoperative pathologic diagnosis, were misdiagnosed as bladder tumors or intramucosal bladder tumors, and only 28.9% were diagnosed prior to surgery (11).

Image analysis and biochemical examinations are crucial for non-functional PUB, as these neoplasms are often found in routine imaging examinations. However, the performance of the tumors are non-specific, making them difficult to distinguish from other bladder tumors. As similar with other types of bladder tumor, conventional imaging studies for bladder paraganglioma often reveal a mural or extramural tumor with wide basilar areas and calcification. Some features that provide clues to bladder paraganglioma include its small intramural lesions that are accentuated in contrast-enhanced MRI and hyperintense on T2 weighted images (12). Metaiodobenzylguanidine (MIGB) is highly specific for functional pheochromocytoma and is often used to distinguish functional and non-functional types. However, this technique is less sensitive than MRI for the detection of paragangliomas (12). The assessment of urinary vanillyl mandelic acid in 24-hour urinary sample contributes to the preoperative diagnosis of functional PUB (13). However, in the absence of the characteristic symptoms of excess CA, these tests are not appreciated by urologists prior to operation. According to previous studies, fluctuating blood pressure and tachyarrhythmia occur in non-functional PUB cases during the TURBT procedure (13). In this study, the blood pressure remained stable at the time of admission and operation. As such, we did not perform a diagnosis of PUB, contrast-enhanced MRI, MIGB examinations, or an investigation of CA metabolites in all patients.

It is difficult to correctly diagnose non-functional PUB pre-operation. Definitive diagnosis is based on histopathology and immunohistochemistry of the excised tumor. From histopathology, paraganglioma cells display characteristic zellballen or nesting patterns with delicate fibrovascular stroma and abundant eosinophilic or amphophilic cytoplasm divided by delicate vascular stroma (10). Similar to the immunophenotypes of other paraganglioma, positive neuroendocrine markers combined with negative epithelial and mesenchymal markers are of significance to its diagnosis. Neuroendocrine markers including CgA, Syn, CD56 and NSE were strongly expressed in the cytoplasm of tumor cells, whilst supporting cells were positively stained for S-100 (1, 14). A metastatic lesion confirmed by pathology is critical to malignant diagnosis. The differential diagnoses of PUB includes urothelial carcinoma, metastatic renal cell carcinoma, prostatic carcinoma, malignant melanoma, and granular cell tumors (10). Histological appearance and immune profiles help to distinguish PUB from other differential diagnoses.

At present, there are no uniform standard treatment options for PUB. The most effective therapy for local PUB remains complete resection. Various surgical options are available including transurethral resection, partial cystectomy and radical cystectomy. Specialists have indicated that partial cystectomy is the mainstream treatment for the disease (15). However, endourethral surgeries, including electro-excision and laser resection have been reported for the treatment of non-functional PUB and lead to the same therapeutic effect as partial cystectomy (16). Katiyar et al. (13) reported 2 non-functional PUB patients who underwent TURBT in which no recurrence after follow-up for 6 and 10 months occurred, indicating that TURBT is an optional and effective treatment for non-functional PUB. In contrast, it has been suggested that resection rarely excises all the tumor residue as the neoplasms often invade the submucosa and muscularis of the urinary bladder, leading to potential tumor recurrence (17). In this study, we successfully treated all patients with PUB with TURBT, and most displayed no recurrence or metastasis after long-term follow-up. Therefore, the complete resection of the tumors through the transurethral approach is a curative option for patients with non-functional PUB, and may represent the mainstream future treatment.

In general, PUB is a rare tumor with uncertain biological behavior, but most PUBs have a good prognosis and slow development. However, paraganglioma has a tendency for recurrence and metastasis, and no standard reference of the duration of follow-up has been reported. Although Beilan et al. (15) indicated that a long-term follow-up is not necessary in benign and local PUB, it has been reported that the rate of local recurrence ranges from 5 to 15%, and that metastasis can occur 20 to 40 years post-surgery (7, 13, 18). As such, Katiyar et al. (13) argued that even non-functional cases should be followed up for an extended time period. In this study, all patients had regular follow-up and only a single recurrence occurred. Despite this, we recommend that long-term periodical CA/metabolite testing, and image-based examinations are performed since the prognosis of PUB remains poorly established.

The limitation of this study includes the lack of case-control studies owing to the unique nature of PUB. However, we are confident that the study holds significance for urologists and enhances our understanding of the diagnosis and treatment of non-functional PUB.

## CONCLUSIONS

Despite its controversial nature, complete transurethral resection of bladder tumor is a safe and curative approach that serves both diagnostic and therapeutic purposes, and avoids the need for partial or radical cystectomy. Histopathological examination and immunohistochemistry are mandatory for a definitive diagnosis, and confirmation of a metastatic lesion through pathology provides the only definite evidence of malignancy in paraganglioma. Regular follow-up through CA and metabolite testing, combined with image-based examinations are recommended to fully eliminate recurrence in these patients.

## COMPLIANCE WITH ETHICAL STANDARDS

The research was approved by the Research Ethics Committee of the Second Hospital of Tianjin Medical University. Informed consents were obtained from the participants. The leader of the Second Hospital of Tianjin Medical University and the ethics committees made an agreement on this research and approved this consent procedure.
